# Analysis of *KRAS* and *BRAF* genes mutation in the central nervous system metastases of non-small cell lung cancer

**DOI:** 10.1007/s10238-015-0349-2

**Published:** 2015-04-23

**Authors:** Marcin Nicoś, Paweł Krawczyk, Bożena Jarosz, Marek Sawicki, Justyna Szumiłło, Tomasz Trojanowski, Janusz Milanowski

**Affiliations:** Department of Pneumonology, Oncology and Allergology, Medical University of Lublin, Jaczewskiego 8, 20-954 Lublin, Poland; Postgraduate School of Molecular Medicine, Medical University of Warsaw, 02-091 Warsaw, Poland; Pathological Laboratory, Department of Neurosurgery and Pediatric Neurosurgery, Medical University of Lublin, 20-954 Lublin, Poland; Department of Thoracic Surgery, Medical University of Lublin, 20-954 Lublin, Poland; Department of Pathomorphology, Medical University of Lublin, 20-954 Lublin, Poland

**Keywords:** NSCLC, Central nervous system metastases, *KRAS* mutations, *BRAF* mutations

## Abstract

*KRAS* mutations are associated with tumor resistance to EGFR TKIs (erlotinib, gefitinib) and to monoclonal antibody against EGFR (cetuximab). Targeted treatment of mutated *RAS* patients is still considered as a challenge. Inhibitors of c-Met (onartuzumab or tiwantinib) and MEK (selumetinib—a dual inhibitor of MEK1 and MEK2) signaling pathways showed activity in patients with mutations in *KRA*S that can became an effective approach in carriers of such disorders. *BRAF* mutation is very rare in patients with NSCLC, and its presence is associated with sensitivity of tumor cells to BRAF inhibitors (vemurafenib, dabrafenib). In the present study, the frequency and type of *KRAS* and *BRAF* mutation were assessed in 145 FFPE tissue samples from CNS metastases of NSCLC. In 30 patients, material from the primary tumor was simultaneously available. Real-time PCR technique with allele-specific molecular probe (KRAS/BRAF Mutation Analysis Kit, Entrogen, USA) was used for molecular tests. *KRAS* mutations were detected in 21.4 % of CNS metastatic lesions and in 23.3 % of corresponding primary tumors. Five mutations were identified both in primary and in metastatic lesions, while one mutation only in primary tumor and one mutation only in the metastatic tumor. Most of mutations were observed in codon 12 of *KRAS*; however, an individual patient had diagnosed a rare G13D and Q61R substitutions. *KRAS* mutations were significantly more frequent in adenocarcinoma patients and smokers. Additional analysis indicated one patient with rare coexistence of *KRAS* and *DDR2* mutations. *BRAF* mutation was not detected in the examined materials. *KRAS* frequency appears to be similar in primary and CNS.

## Introduction

Activating mutations in the mitogen-activated protein kinase (MAPK) pathway, which incorporates the enzymes RAS (rat sarcoma, encoded by *HRAS*, *NRAS* and *KRAS* genes), RAF (rapidly accelerated fibrosarcoma, encoded by *ARAF*, *BRAF* and *CRAF* genes), MEK (MAPK/extracellular-signal-regulated kinase—ERK, encoded by *MEK1* and *MEK2* genes), result in constitutive signaling that leads to oncogenic cell proliferation and cells escape from apoptosis [1, 2].

Kirsten rat sarcoma viral oncogene (KRAS) is involved in proper stimulation of MAPK and PI3-K signaling cascades [1–4]. It was previously described that the *KRAS* gene mutations lead to uncontrolled activation of RAS protein by accumulation of mediators in GTP-binding site [2, 4, 5]. Majority of the *KRAS* gene abnormalities has a missense character located at codons 12, 13 or 61. Occasionally, substitutions in codons 59, 117 and 146 are also reported. The *KRAS* gene mutations have been found above in 40 % of colorectal cancers and in 15–25 % of non-small cell lung cancer (NSCLC)—predominantly in patients with adenocarcinoma and smoking history [2, 4–6].

Clinical trials indicated that the *KRAS* gene mutations are associated with both resistance for reversible EGFR TKIs (epidermal growth factor receptor tyrosine kinase inhibitors: gefitinib, erlotinib) and in reduction of overall survival (OS) in NSCLC patients. For these reasons, the *KRAS* gene mutations are considered as a negative prognostic biomarker in NSCLC patients. Moreover, *KRAS* and *NRAS* genes mutation limits effectiveness of monoclonal antibodies against EGFR (cetuximab, panitumumab) in colorectal cancer patients. [3, 4, 6–8]. Taking into account that RAS protein can activate several signaling pathways, the direct treatment of patients with *KRAS* mutation has proved to be a challenge. However, effectiveness of inhibitors targeted to c-Met (onartuzumab, tiwantinib), MAPK (vemurafenib, dabrafenib) or MEK (trametinib, selumetinib) cascades is promising [4, 6–10].

BRAF serine/threonine protein kinase is involved in sending signals from HER family receptors through RAS protein to transcription factors, which are involved in cell proliferation. About 40–50 % of melanoma patients and a few percent of colorectal cancer patients harbor a mutation in *BRAF* gene, mostly substitution in codon 600. BRAF kinase inhibitors: Vemurafenib and dabrafenib are approved for treatment of late-stage melanoma with *BRAF* mutation. Moreover, in advanced colorectal cancer, *BRAF* mutations are associated with a poor prognosis and possibly resistance to treatment with monoclonal antibodies against EGFR (cetuximab and panitumumab). However, *BRAF* gene mutation is very rare in patients with NSCLC (1–2 %)—mostly in non-smokers with adenocarcinoma histology [1, 11].

To date, the majority of published data evaluated the *KRAS* gene mutations in primary tumors of NSCLC; however, studies assessing these disorders in metastatic lesions are considerably less frequent. For this reason, the main aims of the study were estimation of the incidence of the most common *KRAS* mutations in codons 12, 13 and 61 and *BRAF* V600E substitution in the central nervous system (CNS) metastases in Caucasian patients with advanced NSCLC. Moreover, we performed analysis of differences between molecular profile of metastatic lesions and corresponding primary tumors.

## Materials and methods

### Patients and material

Formalin-fixed, paraffin-embedded (FFPE) tissue samples were enrolled from 145 Caucasian patients with CNS metastases of advanced NSCLC. The corresponding primary NSCLC tumors were simultaneously available from 30 patients. The patients underwent routine neurosurgical procedures with a palliative aim. The median survival time from neurosurgical treatment to death was 9.1 months (information available from 119 patients). All of studied patients were chemotherapy, radiotherapy or molecularly targeted therapies naive. According to number of smoked cigarettes, patients were qualified as heavy smokers (≥15 pack-years), light smokers (<15 pack-years) and non-smokers. Detailed characteristic of studied group has been presented in Table 1.

**Table 1:** Characteristic of studied group

*Gender*	
Male [*n* (%)]	100 (69)
Female [*n* (%)]	45 (31)
*Age*	
Median age ± SD (years)	60 ± 8.8
≥60 years [*n* (%)]	72 (49.7)
<60 years [*n* (%)]	73 (50.3)
*Histopathology*	
Adenocarcinoma [*n* (%)]	80 (55.2)
Squamous-cell carcinoma [*n* (%)]	29 (20)
Large-cell carcinoma [*n* (%)]	22 (15.1)
NSCLC–NOS [*n* (%)]	14 (9.7)
*Smoking status*	
Current smokers [*n* (%)]	73 (50.4)
Former smokers [*n* (%)]	21 (14.5)
Non-smokers [*n* (%)]	36 (24.8)
Lack of data [*n* (%)]	15 (10.3)
*Performance status (PS)*	
0 [*n* (%)]	22 (15.2)
1 [*n* (%)]	76 (52.4)
2 [*n* (%)]	31 (21.4)
3 [*n* (%)]	16 (11)

The study was approved by the ethics committee of the Medical University of Lublin, Poland (No. KE-0254/86/2013).

### Mutation analysis

DNA was isolated from FFPE metastatic tissue samples using QIAamp DNA FFPE Tissue Kit (Qiagen, USA) according to a manufacturer’s protocol. Analysis of the *KRAS* and *BRAF* genes mutation was conducted using real-time PCR equipment (m2000rt, Abbott, USA) with allele-specific, fluorescent and hydrolysis molecular probes (Entrogen, USA). Each probe contains a fluorophore (FAM or VIC) at the 5′-terminus and a quencher at a 3′-terminus. Entrogene KRAS/BRAF Mutations Analysis Kit is able to identify the presence of G12 V, G12C, G12A, G12R, G12D, G12S, G13D, G13S, G13R, G13A, G13C, Q61 K, Q61L, Q61R and Q61H substitutions in *KRAS* gene and V600E substitution in *BRAF* gene. Most samples contain a mixture of wild type (wt) and mutant variants of *KRAS* and *BRAF* genes. The assay is designed to preferentially amplify mutant DNA even in samples with advantage of wt DNA. The assay also amplifies an internal control gene in order to ensure that sufficient amount of DNA is available for amplification. The internal control gene is amplified in all samples, regardless of the presence of a mutation in mentioned genes. Moreover, this Entrogene’s real-time PCR assay is certificated for in vitro diagnosis (CE-IVD), and results obtained in this analysis do not require confirmation using other techniques.

The mutations in *KRAS* and *BRAF* genes were analyzed in total volume of PCR mixture (25 μl) contained: 12.5 μl of Master Mix, 5.9 μl of allele-specific probe, 1 μl of purified genomic DNA (20 ng/μl) and 5.9 μl of nuclease-free water. The amplification of examined region was performed in 96-well plates in following steps: pre-denaturation 95 °C-10 min and 40 cycles in conditions: 95 °C-15 s and 60 °C-40 s. The negative control was determined with DNA isolated from peripheral blood leukocytes of healthy individuals, and the positive control of the analysis was the reaction with control DNA supplied with the assay by the manufacturer.

In our previous published studies, the incidence of mutation in *EGFR* (deletions in exon 19 and substitutions: L858R, T790 M, L861Q, S768I, G719X), *HER2* (A775YVMA or M774AYMVM insertion) and *DDR2* (S768R substitution) genes was assessed in the analyzed material [12–14]. The co-occurrence of these mutations with *KRAS* and *BRAF* genes was also presented in this study.

### Statistical analysis

Statistical analysis was performed using Statistica version 9.0 (Statsoft, USA) and MedCalc 10 (MedCalc software, Belgium). Associations between the occurrence of *KRAS* gene mutations and patient clinical factors were examined using the Chi-square test. The Kaplan–Meier method was used to compare the probability of OS in patients with distinct *KRAS* gene status. Cox regression model with a stepwise selection with minimum AIC factor (Akaike information criterion) was used to assess which of the clinical and genetic factors affect survival. *p* values <0.05 were considered as statistically significant.

## Results

The *KRAS* gene mutations were detected in 21.4 % (31/145) of CNS metastatic lesions of NSCLC. The mutations were frequently (93.5 %, 29/31) observed in codon 12 (15-G12C; 5-G12 V; 3-G12D; 2-G12A; 2-G12S; 2-G12R); however, 2 rare mutations in codons 13 (G13D) and 61 (Q61R) were also detected. In analyzed metastatic samples, we have not detected any V600E substitution in *BRAF* gene.

The *KRAS* gene mutations were significantly more frequent in adenocarcinoma patients than in other types of NSCLC (30 % adenocarcinoma, 6.9 % squamous-cell carcinoma, 13.6 % large-cell carcinoma, 14.3 % not otherwise specified (NOS) NSCLC; *p* = 0.0391; *χ*^2^ = 8.36), in current smokers than in non-smokers and former smokers (22.2 % non-smokers, 42.9 % former smokers, 19.2 % current smokers; *p* = 0.037; *χ*^2^ = 6.567) and in light smokers then heavy smokers (58.8 vs. 16.9 %; *p* = 0.00027; *χ*^2^ = 13.254). On the other hand, there were no differences in the incidence of *KRAS* gene mutations related to gender (20 % women vs. 22.2 % men; *p* = 0.786; *χ*^2^ = 0.074), performance status and age. Clinical characteristics of all patients with detected mutation in the *KRAS* gene have been summarized in Table 2.

**Table 2:** Clinical characteristics of patients with *KRAS* gene mutations

Gender	Diagnosis	Mutation in CNS metastases	Smoking history	Pack-years	OS (mo.)	Age over 60	PS	Primary tumor
Male	Adenocarcinoma	G12C	Never smoker	0	10.2	No	0	mt in CNS tu. and primary tu.
Female	NSCLC–NOS	G12C	Current smoker	60	43	Yes	0	Unavailable
Male	Adenocarcinoma	G12A	Never smoker	0	3.5	Yes	1	Unavailable
Female	Adenocarcinoma	G12C	Current smoker	14	No data	Yes	0	mt in CNS tu., wt in primary tu.
Male	Adenocarcinoma	G12C	Current smoker	40	19.8	Yes	1	Unavailable
Female	NSCLC–NOS	G12C	Former smoker	10	3.8	No	0	Unavailable
Female	NSCLC–NOS	G12A	Current smoker	35	No data	Yes	–	Unavailable
Male	NSCLC–NOS	Q61R	Current smoker	30	0.2	Yes	3	Unavailable
Male	Adenocarcinoma	G12V	Former smoker	20	52.7	Yes	0	mt in CNS tu. and primary tu.
Male	Adenocarcinoma	G12V	Current smoker	40	3.9	Yes	1	Unavailable
Female	Adenocarcinoma	G12C	Former smoker	10	17	No	1	Unavailable
Male	Adenocarcinoma	G12D	Never smoker	0	6.4	Yes	1	Unavailable
Male	NSCLC–NOS	G12C	Former smoker	10	20.4	No	3	Unavailable
Male	Squamous-cell carcinoma	G12C	Never smoker	0	5.8	Yes	0	mt in CNS tu. and primary tu.
Male	Adenocarcinoma	G12C	Current smoker	15	38.3	No	1	mt CNS tu. and primary tu.
Male	NSCLC–NOS	G12S	Never smoker	0	3.8	No	0	Unavailable
Male	NSCLC–NOS	G12R	Former smoker	20	No data	No	1	Unavailable
Male	Large-cell carcinoma	G12S	Current smoker	15	11	No	2	Unavailable
Female	Adenocarcinoma	G12V	Former smoker	10	No data	No	–	Unavailable
Male	Adenocarcinoma	G12D	Former smoker	15	No data	No	–	Unavailable
Male	Adenocarcinoma	G12C	Current smoker	25	36.3	2	2	Unavailable
Male	Adenocarcinoma	G12V	Never smoker	0	19.6	No	1	Unavailable
Female	Squamous-cell carcinoma	G12D	Never smoker	0	No data	Yes	–	Unavailable
Male	Adenocarcinoma	G12C	Current smoker	40	13.6	No	2	Unavailable
Male	Adenocarcinoma	G12C	Current smoker	25	93	No	1	Unavailable
Male	Adenocarcinoma	G13D	Current smoker	50	26.8	Yes	1	Unavailable
Male	Adenocarcinoma	G12C	Current smoker	30	15	No	1	Unavailable
Male	Large-cell carcinoma	G12C	Current smoker	15	18.9	Yes	1	mt in CNS tu. and primary tu.
Male	Adenocarcinoma	G12V	Never smoker	5	12.5	No	1	Unavailable
Female	Adenocarcinoma	G12R	Former smoker	20	6.1	Yes	1	Unavailable
Female	Adenocarcinoma	G12C	Former smoker	0	43.3	No	1	Unavailable
Male	Large-cell carcinoma	wt	Never smoker	0	29.4	Yes	2	wt in CNS tu. and G12C in primary tu.
Male	Large-cell carcinoma	wt	Former smoker	20	4.4	No	2	wt in CNS tu. and G12C in primary tu.

*wt* wild type, *mt* mutant type, *CNS* central nervous system, *tu* tumor

The *KRAS* gene mutations were detected in 23.3 % (7/30) of corresponding primary tumors. However, comparison of molecular profile in matched primary and metastatic lesions indicated some discrepancies. In 5 patients, the *KRAS* gene mutations occurred simultaneously in primary and metastatic lesions, but in 2 patients, the *KRAS* gene mutations (G12C) were detected only in primary tumors. Moreover, in one patient, mutation of the *KRAS* gene (G12C) was observed in metastatic lesion, whereas the status of the *KRAS* gene in corresponding primary tumors was estimated as wild type. We did not detect any mutation in the *BRAF* gene in primary tumors.

In previously study, we found 9 common activating *EGFR* gene mutations (six L858R substitutions and three deletion in exon 19; 6.29 % of studied group), 3 primary T790 M substitution in *EGFR* gene (2.1 %of studied group), three S768R substitutions in *DDR2* gene (2.1 % of studied group) and one insertion in *HER2* gene (0.67 % of studied group) in CNS metastases of NSCLC. The most of CNS metastatic lesions were mutually exclusive. However, in one case we have observed coexistence of S768R mutation in *DDR2* gene with G12C substitution in *KRAS* gene [14].

**Fig. 1:**
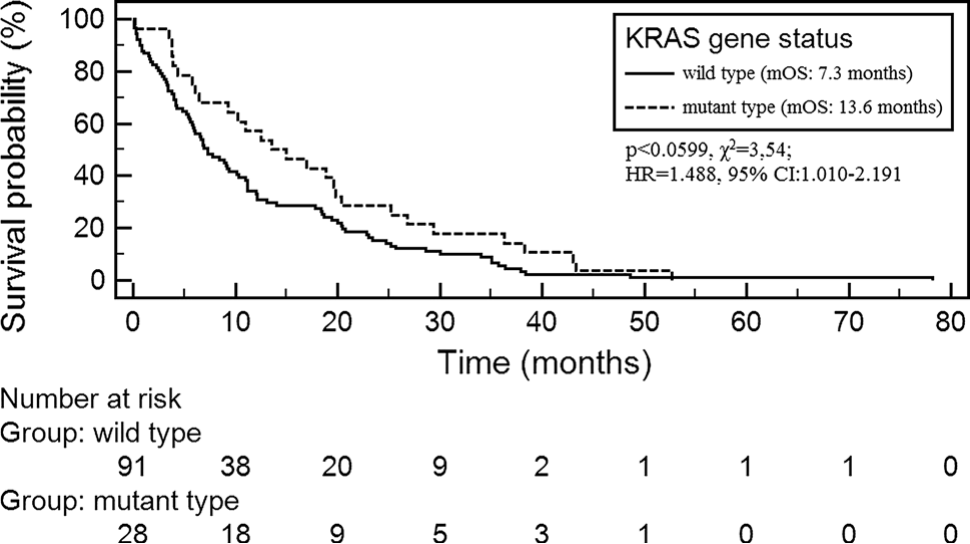
Overall survival probability in NSCLC patients with different status of *KRAS* gene

Demographic and clinical factors did not statistically affect on duration of OS in the studied group. There was also no significant association between median OS (mOS) and the occurrence of the *KRAS* gene mutations. However, patients with the *KRAS* gene mutations had slightly longer mOS than patients without these mutations (13.6 vs. 7.3 months; *p* < 0.0599; *χ*^2^ = 3.54; HR 1.488, 95 % CI 1.010–2.191; Fig. 1).

Cox multivariate logistic regression demonstrated the factors that significantly shortened OS in the studied group (overall model fit: *χ*^*2*^ = 6.703, *p* = 0.035) were as follows: age ≤60 years old (*p* < 0.0499; HR 0.682, 95 % CI 0.466–0.998) and wild-type status of the *KRAS* gene (*p* < 0.0407; HR 0.628, 95 % CI 0.403–0.978).

## Discussion

Brain metastases are one of the most common metastatic lesions of NSCLC, which are associated with high mortality of patients. Till date, we have only limited data concerning evaluation of driver mutations incidence (especially *EGFR* and *KRAS* genes) in CNS metastases of lung cancer. Descriptions of *KRAS* gene mutations in CNS metastatic lesions of NSCLC patients occur only in the form of few case reports and in one large study [15]. For this reason, administration of molecularly targeted therapies for such patients is performed only in single cases [15–17].

### KRAS and BRAF genes mutation frequency in NSCLC patients

In our analysis, the *KRAS* gene mutations were detected in 21.4 % of NSCLC CNS metastases using real-time PCR technique. Villalva et al. [15] using pyrosequencing technique detected the *KRAS* gene mutations in 39 % (30/77) of NSCLC CNS metastases. Moreover, they noticed that pyrosequencing had shown extremely high sensitivity of *KRAS* gene mutations detection in comparison with techniques used in previous studies. However, such highly sensitive tools are not required to reliably identify *KRAS* mutations, and real-time technique with CE-IVD molecular probes is recommended as a satisfying in standard diagnostic procedures [18, 19].

In the studies cited below, *KRAS* gene mutations frequency was analyzed in primary tumors, in metastatic lymph nodes or in available distant metastases. Bauml et al. in group of 374 patients with an informative *KRAS* mutational analysis found 105 (28.1 %) *KRAS* gene mutations. Among 366 patients with informative *EGFR* and *KRAS* mutational analyses, only 1 (0.3 %) patient exhibited both mutations. The frequency of *KRAS* mutations was 20.8 % in male patients and 33.2 % in female patients, 8.3 % in never smokers and 32.7 % in ever smokers, 29.9 % in adenocarcinoma tumors and 20.8 % in other NSCLC tumors [20]. Also in the large study of Kris et al., *KRAS* mutations were the most frequent among other driver mutations in NSCLC patients, and they were found in 182 of 733 analyzed specimens (25 % of patients). Moreover, Kris et al. described 151 *EGFR* mutations (21 %), 57 *ALK* gene rearrangements (8 %), 19 *HER2* mutations (3 %), 16 *BRAF* mutations (2 %), 6 *PIK3CA* mutations (<1 %), 5 *NRAS* mutations (<1 %) and 1 *MEK1* mutation (<1 %) [6]. In European study of Barlesi et al., the 10,000 molecular profiles of NSCLC tumors were characterized. Authors detected 26.9 % tumors with *KRAS* mutations, 9.4 % tumors with *EGFR* mutations, 0.9 % tumors with *HER2* mutations, 1.6 % tumors with *BRAF* mutations and 2.6 % *PI3KCA* mutated tumors as well as 4.0 % tumors with *EML4*–*ALK* fusion genes. Double mutations were seen in 0.9 % of the tumors [22]. It was generally reported that the *KRAS* gene mutations are more frequent in females, smokers and adenocarcinoma subtypes. However, *BRAF* mutation is extremely rare in NSCLC patients [4, 15, 17, 18]. In our study, we indicated the association between the *KRAS* gene mutations presence and smoking status as well as adenocarcinoma diagnosis. However, there was no significant association between the presence of *KRAS* mutations and gender.

Unfortunately, data concerning evaluation of the *KRAS* gene status simultaneously in corresponding metastatic lesions and primary lung carcinomas are limited. In our study, the corresponding primary tumors were available only in 30 patients; however, it remains a considerable group in comparison with previous reports [13, 18–23]. The *KRAS* gene mutations were detected in 7 primary tumors (23.4 %) that was in accordance with Kris, Bauml and Barlesi data obtained in higher groups of patients [6, 20, 21]. Additionally, we observed some discrepancies between molecular profile of metastatic and primary lesions. In 71 % of cases (5/7), the same *KRAS* gene mutations were simultaneously detected in both tumors. However, two mutations were detected only in primary tumors and one only in CNS metastases. Such discrepancies between molecular profile of *EGFR* and *KRAS* genes in corresponding primary tumors and various metastatic lesions had also been reported in previous data [17, 24–28].

Manaco et al. detected 11/40 (27.5 %) of the *KRAS* gene mutations in primary tumors, but the mutations were detected only in 4 (10 %) corresponding metastatic lesions. Moreover, 2 of them had discordant molecular profile of primary and corresponding metastatic lesions [17]. Also Badalian et al. [24] detected three *KRAS* gene mutations both in primary and in metastatic tumors, but only in one case, the mutations were simultaneously observed in both tumors. Schmid et al. reported that the *KRAS* gene mutations were more frequent in primary NSCLC than in metastatic lymph nodes (17 vs. 8 %, respectively). However, in one case different type of the *KRAS* mutation was indicated in primary and metastatic lesions [27]. Similarly, Kalikaki et al. detected the *KRAS* gene mutations in 5/25 of metastatic and primary samples, but concordance between types of mutation in these lesions was observed only in 2 cases. Moreover, they indicated rare coexistence of the *KRAS* gene mutations with deletion in *EGFR* gene that was observed only in primary tumor but not in corresponding metastatic sample [25]. Also Sun et al. [28] described coexistence of the *KRAS* gene mutation with substitution L858R in *EGFR* gene. In previous study, we described one coexistence between S768R substitution in *DDR2* gene with G12C substitution in *KRAS* gene in CNS metastases of NSCLC. Unfortunately, in this patient material from corresponding primary tumor was unavailable. The other CNS metastatic lesions were mutually exclusive [14].

The discordance between mutation presence in metastatic and in their corresponding primary NSCLC tumors suggests that molecular status can be changeable during disease progression. Heterogeneity of primary and metastatic tumors indicated that one tissue sample can be considered as representative for this particular lesion but not for all cancer cells [26, 28, 29]. This knowledge can have a potential clinical implication in qualification of patients for molecularly targeted therapies. However, further studies are required to characterize the correlation with the clinical responses to targeted agents in patients with heterogeneous of the driver mutation status between primary and metastatic lesions [18, 26, 28, 29].

### KRAS gene mutations as a prognostic factor in NSCLC patients

The *KRAS* gene mutations were considered as a negative prognostic factor in NSCLC patients. Clinical outcomes were especially poor in patients with *KRAS* gene mutations after EGFR TKIs therapy [2, 4, 8, 16, 30]. However, clinical trials suggested that the *KRAS* status has no effect on clinical outcomes to EGFR TKIs therapy in patients without *EGFR* gene mutations. In this group of patients, the sensitivity of tumors cell on EGFR TKIs therapy is relatively very low [30, 31].

On the other hand, the LACE-bio study suggested that the *KRAS* gene mutations have no prognostic role in completely resected NSCLC patients treated with adjuvant chemotherapy. However, the overall poor treatment outcomes in the *KRAS* mutation group treated with adjuvant chemotherapy seemed to be caused by the negative predictive value of codon 13 *KRAS* gene mutation [32]. In addition, Moran et al. and Garassiono et al. [33, 34] reported that that tumors with *KRAS* mutations would be more sensitive to pemetrexed. In our study, we observed that patients with mutations in *KRAS* gene had slightly better prognosis than patients with wt *KRAS* gene status. However, our study has a significant weakness. Management of patients after neurosurgery was probably very different, and for this reason, the studied group is very heterogeneous. Lack of data about subsequently used therapies excluded possibilities to evaluate *KRAS* gene status as a reliable prognostic factor.

### Therapy strategies

The presence of the *KRAS* gene mutations is associated with both resistance to EGFR TKIs and reduce of benefits from standard chemotherapy in general group of NSCLC patients [4, 8, 9, 23]. TRIBUTE trial indicated that *KRAS* gene mutations are associated with worse response rate to standard doubled treatment and erlotinib [35]. The INTEREST trial showed that the *KRAS* gene mutations were not a predictive factor for a differential survival effect between gefitinib and docetaxel [36]. The BR.21 and the SATURN trials showed that patients with wt of *KRAS* gene had significant survival benefits from erlotinib in second- or third-line treatment and longer PFS in comparison with patients with *KRAS* gene mutations [37, 38]. Currently, effective RAS inhibitors are not available. However, selumetinib—an oral, selective, non-ATP competitive inhibitor of MEK1/MEK2 kinases and RAF–MEK–ERK (MAPK) inhibitors—can become a new potential agent in personalized NSCLC therapy [7, 9, 22, 23].

Till date, selumetinib monotherapy had shown any clinical benefits in comparison with standard chemotherapy [39–41]. On the other hand, combination of selumetinib and docetaxel demonstrated significant prolongation of PFS in compared to placebo arm (5.3 vs. 2.1 months, respectively). However, differences in median OS (9.4 vs. 5.2 months, respectively) were statistically insignificant. Moreover, the proportion of serious adverse events (especially neutropenia, diarrhea, nausea and vomiting) was higher in the selumetinib group [41]. Similar, results of preclinical in vivo studies had shown that doublet therapy (selumetinib and docetaxel) leads to more effective inhibition and regression of tumor growth [4, 7, 22]. Moreover, ongoing clinical trials show that combination of targeting agents against different signaling pathways may provide additional benefits in treatment of patients with unregulated MEK, MAPK, RAF and RAS pathways. The presence of *BRAF* gene mutation is associated with sensitivity of tumor cells to BRAF inhibitors (vemurafenib, dabrafenib). However, before routine application, they need further studies [4, 10, 23, 41].

## Conclusions

The *KRAS* gene mutations (especially in codon 12) are the most frequent genetic abnormalities both in primary and in CNS metastatic lesions of NSCLC. Moreover, the results of this study have indicated discrepancies between molecular profile of some CNS metastases and corresponding primary tumors that may be caused by acquisition of heterogeneity during disease progression. For this reason, secondary tumors or metastatic sites should be retested for molecular abnormalities due to a relatively high rate of possible alterations. Further studies (especially clinical trials) are needed to characterize the correlation between *KRAS* gene status and clinical outcomes in NSCLC patients.

## Abbreviations

CE-IVD: Certificated for in vitro diagnosis
CNS: Central nervous system
KRAS: Kirsten rat sarcoma
FFPE: Formalin-fixed paraffin-embedded
NOS: Not otherwise specified
NSCLC: Non-small cell lung cancer
OS: Overall survival
PFS: Progression-free survival
RR: Response rate
mt: Mutant type
wt: Wild type
